# Nomogram for predicting the risk of preterm delivery after IVF/ICSI treatment: an analysis of 11513 singleton births

**DOI:** 10.3389/fendo.2023.1065291

**Published:** 2023-05-18

**Authors:** Zhiqi Liao, Lei Cai, Chang Liu, Jie Li, Xinyao Hu, Youhua Lai, Lin Shen, Cong Sui, Hanwang Zhang, Kun Qian

**Affiliations:** ^1^ Reproductive Medicine Center, Tongji Hospital, Tongji Medical College, Huazhong University of Science and Technology, Wuhan, China; ^2^ Reproductive Medicine Center, The Affiliated Drum Tower Hospital of Nanjing University Medical College, Nanjing, China; ^3^ Gynaecology and Obstetrics, The Fourth Affiliated Hospital of ZheJiang University School of Medicine, Yiwu, China

**Keywords:** nomogram, prediction, preterm birth, *in vitro* fertilization, intracytoplasmic sperm injection

## Abstract

**Background:**

There is a higher risk of preterm delivery (PTD) in singleton live births conceived after *in vitro* fertilization (IVF)/intracytoplasmic sperm injection (ICSI) compared with spontaneously conceived pregnancies. The objective of our study was to build a predictive nomogram model to suggest the possibility of PTD in singleton pregnancies after IVF/ICSI treatment.

**Method:**

11513 IVF/ICSI cycles with singleton live births were enrolled retrospectively. These cycles were randomly allocated into a training group (80%) and a validation group (20%). We used the multivariate logistics regression analysis to determine prognostic factors for PTD in the training group. A nomogram based on the above factors was further established for predicting PTD. Receiver operating characteristic curves (ROC), areas under the ROC curves (AUC), concordance index (C-index), and calibration plots were analyzed for assessing the performance of this nomogram in the training and validation group.

**Results:**

There were fourteen risk factors significantly related to PTD in IVF/ICSI singleton live births, including maternal body mass index (BMI) > 24 kg/m^2^, smoking, uterine factors, cervical factors, ovulatory factors, double embryo transferred (DET), blastocyst transfer, FET, vanishing twin syndrome (VTS), obstetric complications (placenta previa, placenta abruption, hypertensive of pregnancies, and premature rupture of membrane), and a male fetus. These factors were further incorporated to construct a nomogram prediction model. The AUC, C-index, and calibration curves indicated that this nomogram exhibited fair performance and good calibration.

**Conclusions:**

We found that the occurrence of PTD increased when women with obesity, smoking, uterine factors, cervical factors, ovulatory factors, DET, VTS, and obstetric complications, and a male fetus. Furthermore, a nomogram was constructed based on the above factors and it might have great value for clinic use.

## Introduction

Infertility is a global medical condition that is estimated to affect 8%-12% of women worldwide (1). An increasing number of infertile couples of reproductive ages seek treatments, and thus, the use of assisted reproductive technology (ART) is climbing. In recent decades, many reproductive clinicians have paid more attention to the safety of offspring in ART (2). Unfortunately, they found a higher incidence of adverse perinatal outcomes in neonates born after *in vitro* fertilization (IVF)/intracytoplasmic sperm injection (ICSI), even in singleton pregnancies, than those conceived naturally (3, 4). It has been reported that preterm delivery (PTD), a leading determinant of neonatal mortality, is also more common in IVF infants than in those conceived with spontaneous conception (5–7).

PTD is defined as delivery before 37 weeks of gestation (8). As mentioned, it is a key cause of newborn deaths, which can bring up new couples’ significant socioeconomic burden (5, 6, 8). There is still no consensus on which factors are associated with the increasing risk of PTD in IVF/ICSI cycles (9). In an analysis of 144018 IVF cycles, many patients’ inherent risk factors, such as infertility duration, cause of infertility, and donor oocytes, were responsible for a higher incidence of PTD (10). After that, in a study analyzing more than 20,000 singleton infants revealed that blastocyst transfer and frozen-thawed embryo transfer (FET) were also risk factors for PTB in addition to female inherent risk factors (11). Besides, a meta-analysis conducted by Pinborg et al. showed that PTD was also related to IVF procedures (12). Thereinto, researchers have demonstrated that IVF singletons had an increasing risk of PTD compared to ICSI singletons (12). However, data available by Keyhan et al. did not support this finding (9). No increasing risk of PTD was observed by Keyhan et al. between IVF babies and ICSI babies after propensity score matching based on baseline characteristics (9). This inconsistency highlights the need for further exploring the related risk factors for PTD in IVF/ICSI singletons. It is also important to construct a predictive model based on these factors, which enables clinicians to predict the occurrence of PTD, allowing for early implementation of interventions to prevent the onset of it.

Nomogram is an intuitive graph of a predictive model, which has been used to predict incidence or prognosis of diseases by integrating important prognostic factors (13, 14). It is helpful for us to create an easy-to-use predictive model for assessing the incidence of PTD in IVF/ICSI singletons. In this study, we aimed to analyze the hazardous factors of PTD to construct a nomogram predictive model for screening the risk of PTD in IVF/ICSI pregnancies.

## Patients and methods

### Patient enrollment

From January 2015 to February 2021, IVF/ICSI cycles with live births at the Reproductive Medicine Center of Tongji Hospital were retrospectively identified. Inclusion criteria were shown below: a) cycles resulting in singleton births; b) fresh and FET cycles; c) cycles with gonadotropin releasing hormone (GnRH) stimulation protocols in fresh cycles; d) natural cycles and cycles with hormone replacement in FET cycles. Moreover, exclusion criteria were as follows: a) cycles with donor eggs; b) cycles with non-gonadotropin releasing hormone (GnRH) stimulation protocols; c) cycles with preimplantation genetic diagnosis; d) cycles with missing baseline and follow-up data. All information was retrieved from the electronic medical record system. This study was approved by the Medical Ethics Committee of Tongji Hospital, Tongji Medical College, affiliated Huazhong University of Science and Technology (TJ-IRB20220624).

### Controlled ovarian stimulation (COS) protocols in fresh cycles

As mentioned above, fresh cycles with GnRH stimulation protocols were included in our analysis. In light of ovarian reserve, female age, and other characteristics, the individualized COS protocol for each patient was determined. Generally, women with a normal ovarian response were provided with a long GnRH agonist (GnRH-a) regimen, while a flexible GnRH antagonist (GnRH-ant) protocol was offered to women with a diminished ovarian reserve or a known poor response (15). There are four COS protocols, including the GnRH-a ultralong protocol, the depot GnRH-a protocol, the GnRH-a long protocol, and the GnRH-ant protocol. All these protocols were described previously (16–18).

In short, in the GnRH ultralong protocol, women were injected subcutaneously with 3.75 mg long-acting GnRH-a (Decapeptyl; Ferring, SaintPrex, Switzerland) on days 1-3 of cycles to achieve pituitary suppression (16). The depot GnRH-a protocol was performed by subcutaneously administering 3.75 mg long-acting GnRH-a (Decapeptyl; Ferring, Saint-Prex, Switzerland) on Day 2 of the cycle. Moreover, in the GnRH-a long protocol, patients were provided with a daily subcutaneous injection of 0.1 mg GnRH-a (Decapeptyl; Ferring, Saint-Prex, Switzerland), initiating from the mid-luteal phase. In GnRH-a protocols, when complete pituitary desensitization was confirmed (a low serum estradiol (E_2_) level of ≤30 pg/mL and a serum luteinizing hormone level of ≤ 2 IU/L), gonadotropins (Gn) were applied. While in the GnRH-ant protocols, the stimulation process starts with the administration of recombinant follicle stimulating hormone (r-FSH) (Gonal-F; Merck-Serono, Geneva, Switzerland) from Day 2 or 3 of the cycle. Then, the GnRH-ant cetrorelix acetate (Cetrotide; Merck-Serono) was injected at 0.25 mg/d to prevent premature ovulation when one of the criteria was met (E_2_ levels >300 pg/ml, LH levels >10 IU/l, and the diameter of at least one follicle >14 mm).

When two leading follicles reached a mean diameter of 18 mm or three follicles reached a mean diameter of 17 mm, 250 µg human chorionic gonadotrophin (hCG) (Ovidrel; Merck-Serono, Geneva, Switzerland) was used to trigger ovulation. Oocytes were retrieved 34-36 h after triggering.

### IVF/ICSI-embryo transfer (ET) procedures

The processes of oocyte fertilization, embryo culture, and embryo transfer have been described previously (15, 16). In brief, oocytes were fertilized by IVF, ICSI, or rescue ICSI (RICSI). RICSI was performed when there was no second polar body (PB) in oocytes or <25% of the oocytes presented a second PB after insemination with IVF (19). Normal fertilization was defined as the appearance of two pronuclei (2PN). Moreover, a maximum of two embryos were transferred.

### The quality of embryos

On day 3 (D3) after oocyte retrieval, all of the cleavage embryos were checked. Good-quality embryos on D3 comprised seven to eight blastomeres without multinucleation and had < 20% fragments (16). The quality of blastocysts (D5 or D6) was evaluated using the Gardner Blastocyst Scoring System (20). In short, according to the cell number and junction, the inner cell mass and trophectoderm of blastocysts were graded from A to C. Good-quality blastocysts were defined as AA, AB, BA, and BB (20).

### Embryo freezing and recovery

Based on the instructions of the vitrified freezing/resuscitation solution (Japan Kato), the embryos were frozen and thawed. The survival of thawed embryos was observed after 2H. The surviving cleavage embryos reached or exceeded half of the blastomere, while the surviving blastocyst partially or fully dilated (21).

### Endometrial preparation in FET cycles

The endometrial preparation was described previously (21). In brief, in the natural cycles, the follicle, endometrium and ovulation were monitored during the menstrual cycle. When the peak of luteinizing hormone (LH) was observed, progesterone (P) was given. The cleavage embryos and blastocysts were thawed on D3 and D5 respectively after ovulation. As for the hormone replacement cycles, estradiol valerate (Bayer, Germany) was taken orally from D2 to D12 during the menstrual cycle. When the endometrium was ≥7 mm and the estrogen action time was ≥10 days, P was administrated. The cleavage embryos and blastocysts were thawed on D4 and D6 respectively after ovulation.

### Data collection

Data were collected on female age, female BMI, women smoking, duration of infertility (years), antral follicle counts (AFC), baseline FSH, type of infertility (primary or secondary infertility), and infertility factors (tubal, pelvic, endometriosis, uterine, cervical, ovulatory, diminished ovarian reserve (DOR), male, or unexplained factors) in fresh or FET cycles.

In fresh cycles, the following factors were included in the analysis: stimulation protocols (GnRH-a ultralong, GnRH-a depot, GnRH-a long, or GnRH-ant protocols), days of Gn, total dose of Gn, E_2_ and P level on the triggering day, endometrial thickness (EMT) on the triggering day, No. of oocyte retrieved, type of fertilization (IVF, ICSI, or RICSI), No. of embryo transferred (double or single embryo transferred, DET or SET), type of embryo (cleavage embryo or blastocyst), and morphology of the embryos transferred.

In FET cycles, the factors as follows were included: type of endometrial preparation, such as natural cycles and hormone replacement cycles (HRT), EMT on progesterone administration, type of fertilization (IVF, ICSI, or RICSI), DET or SET, type of embryo (cleavage embryo or blastocyst), and morphology of the embryos transferred.

During pregnancies, gestational sacs, obstetric complications, such as placenta previa (PP), placenta abruption (PA), hypertensive of pregnancies (HDP), or premature rupture of membrane (PROM), neonatal gender, birth weight and gestational age were all collected. In our study, PTD was defined as delivery with gestational age reached 28 weeks but less than 37 weeks. All relevant information in the electronic medical record system was collected and checked by two authors (Z.L. and L.C.).

### Nomogram

All selected risk factors were enrolled to establish the nomogram model using the R package “rms”. The predictive model was calculated using the R package “lrm” and displayed as an interactive nomogram by “regplot”. The multivariate logistic regression coefficient (β) generated a risk score with each factor. Each factor had its point (corresponding line is β(X-m) terms), and the total scores were calculated by adding all the points together. The total scores corresponded to the probability of PTD occurring.

### Statistical analysis

In this study, continuous data with normal distribution are presented as the mean ± SD, while non-normal distribution data are displayed as median (IQR). Categorical data are presented as numbers (percentages). T tests or Mann‐Whitney U tests were utilized to compare the continuous data, while chi-square tests or Fisher’s exact tests were used to analyze the categorical data. Univariate values with P value < 0.1 were screened for the next step of multivariate regression analysis. In the multivariate logistic regression analysis, adjusted odds ratios (AORs) and 95% confidence intervals (CIs) were used to indicate the level of association between risk factors and PTD. P value < 0.05 was considered statistically significant. SPSS 26 software was used for statistical analysis.

All statistically significant risk factors in the multivariate logistic regression analysis were further used to build a nomogram predictive model. The discrimination calibration abilities of this model were evaluated by calculating receiver operating characteristic curves (ROC), area under the ROC curve (AUC), Harrell’s concordance index (C-index), and the calibration plots in the training and validation groups (14). The value of the C-index and AUC ranges from 0 to 1, with 0.5-0.7 indicating poor performance of this model, 0.7-0.8 demonstrating fair performance, and 0.8-0.9 suggesting good performance (22). R software (version 4.0.5) was used to construct the nomogram model and analyze the ROC, AUC, C-index, and calibration curve.

## Results

### General characteristics of patients

**Figure 1:**
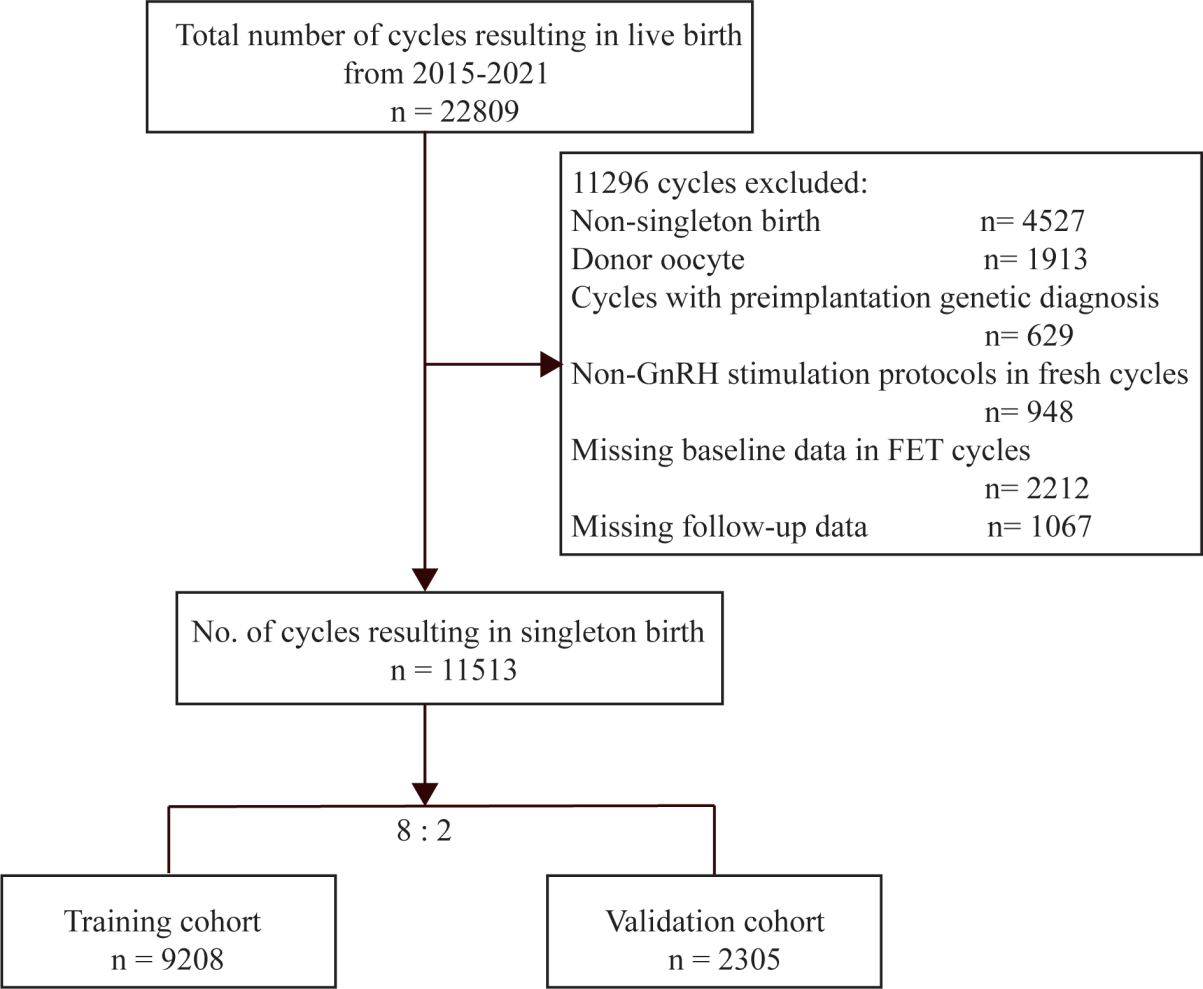
Flow chart of data selection.

A total of 22809 cycles resulting in live births were identified (**Figure 1**). Among these cycles, 11296 cycles were not available for analysis, as one of the following exclusion criteria was met: (a) multiple live births (4527cycles), (b) donor oocyte (1913 cycles), (c) non-GnRH stimulation protocols in fresh cycles (948 cycles), (d) cycles with preimplantation genetic diagnosis (629 cycles), and (e) missing baseline or follow-up data (3279 cycles). Finally, the number of IVF/ICSI available for analysis was 11513. Then, we randomly allocated 9208 cycles (80%) to the training group and 2305 cycles (20%) to the validation group.

**Table 1** described the baseline characteristics of the IVF/ICSI cycles with singleton live births between the training group and validation group. No significant difference in most characteristics was observed between the two groups (**Table 1**). Furthermore, **Table 2** demonstrated a comparison of baseline characteristics between the PTD and non-PTD groups in the training group. There were 953 cycles (10.3%) in the PTD group and 8255 cycles (89.7%) in the non-PTD group (**Table 2**). As shown in **Table 2**, the median (IQR) of birth weight was 3050 (1750) and 2060 (1210) in non-PTD and PTD group respectively. The incidence of PTD was higher if there was one of the following factors: (a) female age ≥ 35, (b) female BMI > 24 kg/m^2^, (c) smoking, (d) high baseline FSH, (e) secondary infertility, (f) uterine factors, (g) cervical factors, (h) ovulatory factors, (i) EMT ≤7 mm, (j) two embryo transferred, (k) blastocyst transfer, (l) FET, (m) non-high quality embryo transfer, (n) two gestational sacs, (o) obstetric complications (PP, PA, GDMHDP, and PROM), and (p) a male fetus (**Table 2**).

**Table 1:** Demographic characteristic of patients in the training and validation groups.

Variables	Training group n=9208	Validation group n = 2305	P value
Female age, y			.626
<35	7772 (84.4%)	1955 (84.8%)	
≥35	1436 (15.6%)	350 (15.2%)	
Female BMI, kg/m^2^			.824
≤24	6674 (72.5%)	1676 (72.7%)	
>24	2534 (27.5%)	629 (27.3%)	
Smoking	119 (1.3%)	30 (1.3%)	.972
Duration of infertility^#^, y	3.00 (2.00)	3.00 (2.00)	.971
AFC			.457
5-24	7993 (86.8%)	2005 (87.0%)	
<5	410 (4.5%)	90 (3.9%)	
≥24	805 (8.7%)	210 (9.1%)	
Baseline FSH^#^, mIU/ml	7.21 (2.29)	7.14 (2.27)	.333
Type of infertility			
Primary	6094 (66.2%)	1543 (66.9%)	.490
Secondary	3114 (33.8%)	762 (33.1%)	
Factors of infertility			
Tubal Pelvic Endometriosis Uterine Cervical Ovulatory DOR Male Unexplained	4924 (53.5%) 1724 (18.7%) 866 (9.4%) 1779 (19.3%) 61 (0.7%) 2162 (23.5%) 1160 (12.6%) 3429 (37.2%) 527 (5.7%)	1191 (51.7%) 441 (19.1%) 216 (9.4%) 435 (18.9%) 16 (0.7%) 558 (24.2%) 264 (11.5%) 895 (38.8%) 140 (6.1%)	.120 .653 .960 .625 .867 .461 .136 .159 .520
EMT, mm			
≤7	171 (1.9%)	43 (1.9%)	.979
>7	9037 (98.1%)	2262 (98.1%)	
Treatment type			
IVF ICSI IVF+RICSI	6095 (66.2%) 2607 (28.3%) 506 (5.5%)	1496 (64.9%) 703 (30.5%) 106 (4.6%)	.042*
No. of embryo transferred			.612
1	5801 (63.0%)	1439 (62.4%)	
2	3407 (37.0%)	866 (37.6%)	
Blastocyst transfer	4085 (44.4%)	1036 (44.9%)	.615
FET	4037 (43.8%)	992 (43.0%)	.486
High-quality embryo transfer	7548 (82.0%)	1890 (82.0%)	.979
Gestational sacs			.129
1	8364 (90.8%)	2070 (89.8%)	
2	844 (9.2%)	235 (10.2%)	
PP	282 (3.1%)	65 (2.8%)	.542
PA	89 (1.0%)	22 (1.0%)	.958
GDM	454 (4.9%)	136 (5.9%)	.059
HDP	279 (3.0%)	55 (2.4%)	.100
PROM	127 (1.4%)	31 (1.3%)	.899
Neonatal gender			.885
Male	5018 (54.5%)	1260 (54.7%)	
Female	4190 (45.5%)	1045 (45.3%)	
Birth weight, g	2950 (1725)	3000 (1727)	.979
PTD	953 (10.3%)	223 (9.7%)	.339

BMI, body mass index; AFC, antral follicles count; FSH, follicle-stimulating hormone; DOR, diminished ovarian reserve; EMT, endometrial thickness; IVF, in vitro fertilization; ICSI, intracytoplasmic sperm injection; RICSI, rescue ICSI; FET, frozen-thawed embryo transfer; PP, placenta previa; PA, placenta abruption; GDM, gestational diabetes mellitus; HDP, hypertensive of pregnancies; PROM, premature rupture of membrane; PTD, preterm delivery; *P<0.05. #: The data were presented as median (IQR).

**Table 2:** Baseline characteristics of patients with singleton live birth between PTD and non-PTD groups in the training group.

Variables	Non-PTD n = 8255	PTD n = 953	P value
Female age, y			
<35	6992 (84.7%)	780 (81.8%)	.022*
≥35	1263 (15.3%)	173 (18.2%)	
Female BMI, kg/m^2^			.082*
≤24	6006 (72.8%)	668 (70.1%)	
>24	2249 (27.2%)	285 (30.0%)	
Smoking	78 (0.9%)	41 (4.3%)	<.001*
Duration of infertility^#^, y	3.00 (3.00)	3.00 (2.00)	.634
AFC			.699
5-24	7173 (86.9%)	820 (86.0%)	
<5	363 (4.4%)	47 (4.9%)	
≥24	719 (8.7%)	86 (9.0%)	
Baseline FSH^#^, mIU/ml	7.06 (2.34)	7.23 (2.28)	.013*
Type of infertility			.053*
Primary	5490 (66.5%)	604 (63.4%)	
Secondary	2765 (33.5%)	349 (36.6%)	
Factors of infertility			
Tubal Pelvic Endometriosis Uterine Cervical Ovulatory DOR Male Unexplained	4418 (53.5%) 1538 (18.6%) 770 (9.3%) 1541 (18.7%) 49 (0.6%) 1902 (23.0%) 1031 (12.5%) 3101 (37.6%) 479 (5.8%)	506 (53.1%) 186 (19.5%) 96 (10.1%) 238 (25.0%) 12 (1.3%) 260 (27.3%) 129 (13.5%) 328 (34.4%) 48 (5.0%)	.804 .507 .455 <.001* .016* .003* .356 .057* .335
EMT, mm			.035*
≤7 >7	145 (1.8%) 8110 (98.2%)	26 (2.7%) 927 (97.3%)	
Treatment type IVF ICSI IVF+RICSI	5444 (65.9%) 2351 (28.5%) 460 (5.6%)	651 (68.3%) 256 (26.9%) 46 (4.8%)	.305
No. of embryo transfer			<.001*
1	5283 (64.0%)	518 (54.4%)	
2	2972 (36.0%)	435 (45.6%)	
Blastocyst transfer	3501 (42.4%)	584 (61.3%)	<.001*
FET	3445 (41.7%)	592 (62.1%)	<.001*
High-quality embryo transfer	6822 (82.6%)	726 (76.2%)	<.001*
Gestational sacs 1 2	7665 (92.9%) 590 (7.1%)	699 (73.3%) 254 (26.7%)	<.001*
PP	220 (2.7%)	62 (6.5%)	<.001*
PA	34 (0.4%)	55 (5.8%)	<.001*
GDM	402 (4.9%)	52 (5.5%)	.428
HDP	179 (2.2%)	100 (10.5%)	<.001*
PROM	45 (0.5%)	82 (8.6%)	<.001*
Neonatal gender			.003*
Male	4455 (54.0%)	563 (59.1%)	
Female	3800 (46.0%)	390 (40.9%)	
Birth weight^#^, g	3050 (1750)	2060 (1210)	<.001*

PTD, preterm delivery; BMI, body mass index; AFC, antral follicles count; FSH, follicle-stimulating hormone; DOR, diminished ovarian reserve; EMT, endometrial thickness; IVF, in vitro fertilization; ICSI, intracytoplasmic sperm injection; RICSI, rescue ICSI; FET, frozen-thawed embryo transfer; PP, placenta previa; PA, placenta abruption; GDM, gestational diabetes mellitus; HDP, hypertensive of pregnancies; PROM, premature rupture of membrane; #, data are presented as median (interquartile range, IQR); *P<0.1.

### Clinical characteristics in fresh cycles

When only included fresh cycles, we also found the difference in female age, female BMI, smoking, cervical factors, ovulatory factors, EMT, gestational sacs, obstetric complications, and neonatal gender between PTD and non-PTD groups (**Table S1**). In addition to the patient’s characteristics, data related to ovarian stimulation in the fresh cycle were also presented in **Table S1**, including stimulation protocols, average dose of Gn, E2 or P on hCG day, and No. of oocyte retrieved. The result showed that women with GnRH-agonist long protocol had lower risk to deliver a preterm baby, while women with GnRH-antagonist protocol had higher risk (**Table S1**).

### Clinical characteristics in FET cycles

When only included FET cycles, there was also a higher risk for PTD in women with obesity, uterine factors, DET, non-high quality embryo transfer, two gestational sacs, obstetric complications, and neonatal gender between PTD and non-PTD groups (**Table S2**). However, no difference was observed in the type of endometrial preparation (natural cycles and HRT cycles) between PTD and non-PTD groups (**Table S2**).

### Logistic regression analysis

To eliminate confounding factors, multivariate logistic regression analysis was used to analyze the risk factors for PTD, which were detailed in **Table 3**. Factors (female age, female BMI, smoking, baseline FSH, type of infertility, uterine factors, cervical factor, ovulatory factor, male factor, EMT, No. of embryo transferred, blastocyst transfer, FET, high-quality embryo transfer, gestational sacs, PP, PA, GDM, HDP, PROM, and neonatal gender) with P<0.1 in **Table 2** were entered into the multivariate logistic regression analysis. Thirteen factors remained statistically significant. Although P value in cervical factor was 0.053 (P>0.05), we still included this factor in further analysis as this factor had very important clinical significance in the occurrence of PTD.

**Table 3:** Multivariable logistic regression model for predicting occurrence of PTD in training group.

Factors	AOR (95%CI)	P value
Female age		.344
<35	1	
≥35	1.10 (0.90-1.34)	
Female BMI		.010*
≤24	1	
>24	1.25 (1.06-1.47)	
Smoking	4.97 (3.21-7.67)	<.001*
Type of infertility		.373
Primary	1	
Secondary	1.08 (0.92-1.26)	
Basal FSH	1.01 (0.97-1.04)	.695
Uterine factors	1.47 (1.23-1.76)	<.001*
Cervical factors	2.02 (0.99-4.11)	.053
Ovulatory factors	1.24 (1.04-1.47)	.016*
Male factors	0.98 (0.84-1.15)	.791
EMT		.305
≤7	1	
>7	1.28 (0.80-2.06)	
No. of embryo transfer		<.001*
1	1	
2	1.37 (1.17-1.61)	
Blastocyst transfer	1.56 (1.19-2.03)	.001*
FET	1.56 (1.20-2.03)	.001*
High-quality embryo transfer	0.88 (0.74-1.06)	.168
Gestational sacs		<.001*
1	1	
2	4.17 (3.46-5.03)	
PP	3.06 (2.24-4.19)	<.001*
PA	10.22 (6.35-16.46)	<.001*
HDP	4.26 (3.21-5.66)	<.001*
PROM	20.32 (13.55-30.47)	<.001*
Neonatal gender Male Female	1.182 (1.019-1.371) 1	.027*

PTD, preterm delivery; BMI, body mass index; FSH, follicle-stimulating hormone; EMT, endometrial thickness; FET, frozen-thawed embryo transfer; PP, placenta previa; PA, placenta abruption; HDP, hypertensive of pregnancies; PROM, premature rupture of membrane; AOR, adjusted odds ratio; CI, confidence interval; *P<0.05.

The results showed that compared to women whose BMI was ≤ 24 kg/m2, overweight patients had a significantly higher risk of PTD after IVF/ICSI (OR 1.25, 95% CI: 1.06-1.47). Women who had a smoking habit were more likely to deliver a preterm infant (OR 4.97, 95% CI: 3.21-7.67). Moreover, women with uterine, cervical, and ovulatory infertility factors displayed a rising PTD ratio (OR 1.47, 95% CI: 1.23-1.76; OR 2.02, 95% CI: 0.99-4.11; OR 1.24, 95% CI: 1.04-1.47, respectively). In comparison to the SET group, women who had DET had a higher risk of PTD (OR 1.37, 95% CI: 1.17-1.61). Besides, women with blastocyst transfer (OR 1.56, 95% CI: 1.19-2.03) and FET (OR 1.56, 95% CI:1.20-2.03) had higher risk of PTD. During pregnancies, women who had two gestational sacs had a greater chance of delivering a preterm baby (OR 4.17; 95% CI: 3.46-5.03). In addition, women who had obstetric complications, such as PP (OR 3.06, 95% CI: 2.24-4.19), PA (OR 10.22, 95% CI: 6.35-16.46), HDP (OR 4.26, 95% CI: 3.21-5.66), and PROM (OR 20.32, 95% CI: 13.55-30.47), also had an increasing risk of PTD. Interesting, we found a male fetus (OR 1.182, 95% CI: 1.019-1.371) was more likely to be born prematurely (**Table 3**).

### Nomogram prediction model

**Figure 2:**
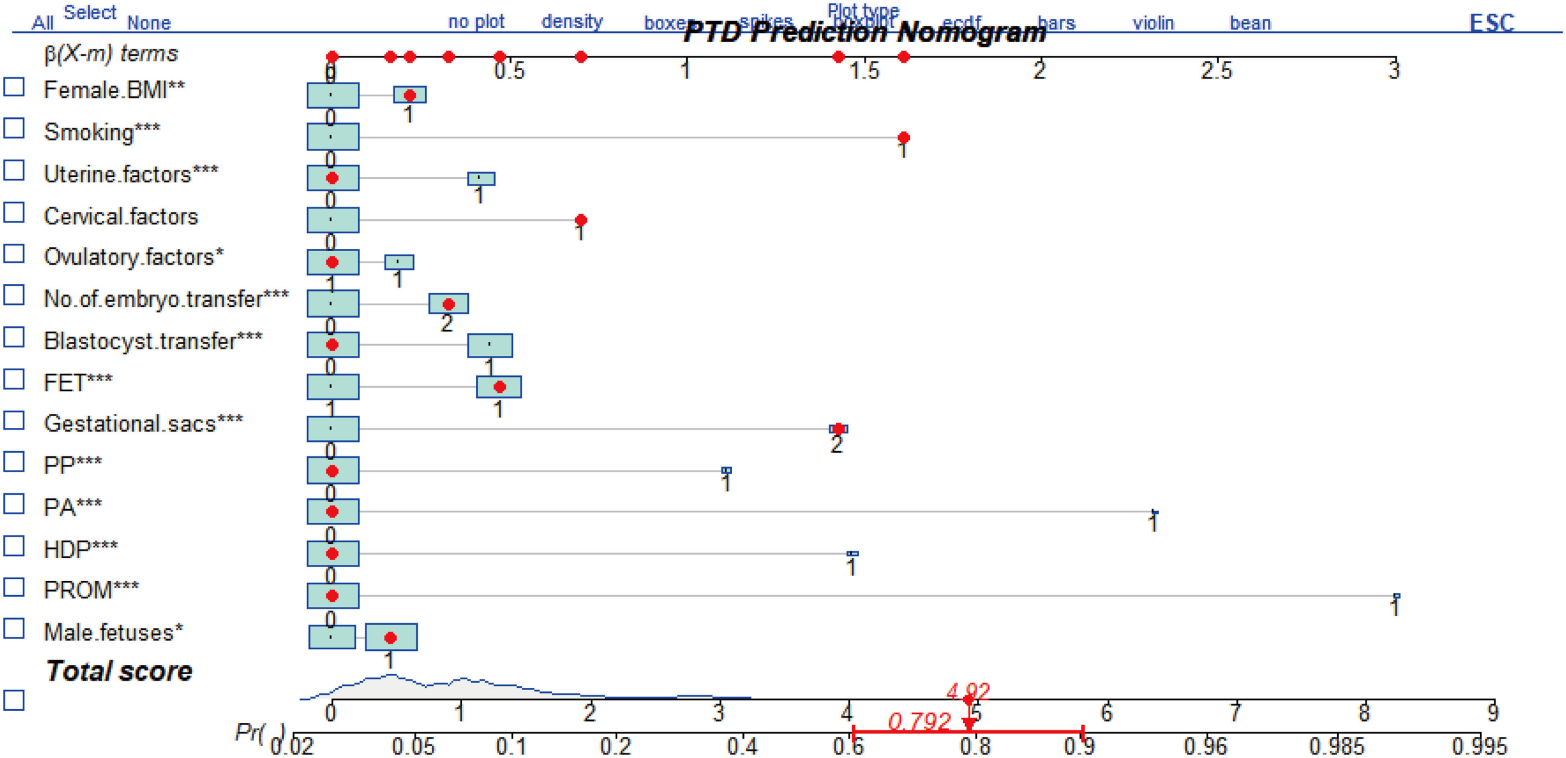
The nomogram to predict the incidence of PTD in patients after IVF/ICSI. It includes female body mass index (BMI), smoking, uterine factors, cervical factors, ovulatory factors, No. of embryo transferred, blastocyst transfer, frozen-thawed embryo transfer (FET), gestational sacs, placenta previa (PP), placenta abruption (PA), hypertensive of pregnancies (HDP), and premature rupture of membrane (PROM). The usage of this nomogram is interpreted in an assumptive woman with BMI >24 kg/m^2^, smoking habit, cervical factors, two embryo transfer (DET), FET, two gestational sacs, and a male fetus. For this woman, the total point added up to 4.92, which indicated approximately 0.792 of probability of PTD incidence. *P<0.05, **P<0.01, ***P<0.001.

Furthermore, the above prognostic factors were incorporated to establish a predictive model for predicting the probability of PTD incidence in IVF/ICSI singleton live birth (**Figure 2**). The nomogram developed from the training group was shown in **Figure 2**. The usage of the nomogram is illustrated with an assumptive woman with BMI > 24, smoking, cervical factor, DET, FET, two gestational sac, and have a male fetus. The total score added up to 4.92 for this patient, which represents approximately 0.792 of probability of PTD incidence (**Figure 2**).

### Validation of the nomogram model

**Figure 3:**
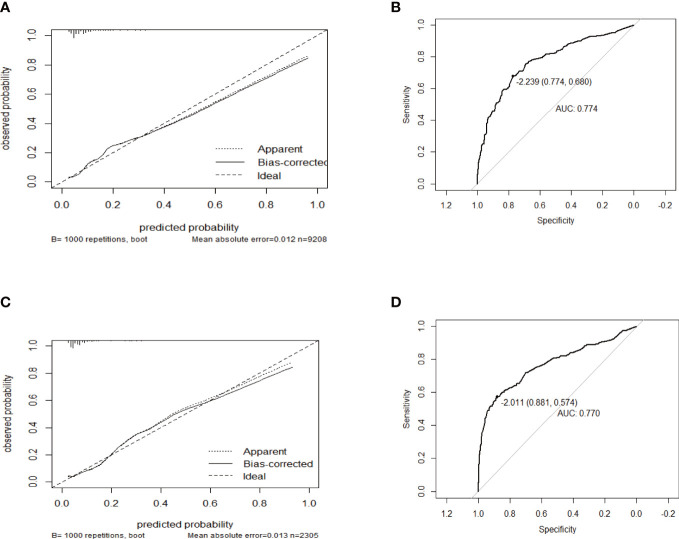
The calibration plots and receiver operator characteristic curves (ROC) of the nomogram in the training and validation group. Calibration plots in **(A)** the training group and **(C)** validation group. ROC in **(B)** the training group and **(D)** validation group.

The performance of this nomogram was assessed by AUC, C-index, and calibration plots. In the training and validation groups, both calibration plots indicated good agreement between the predicted probability and actual observation of PTD (**Figures 3A, C**). In addition, the AUC for this predictive model was 0.774 and 0.770 in the training and validation groups, respectively (**Figures 3B, D**). The optimal threshold was -2.239 and -2.011 in training and validation groups, respectively (**Figures 3B, D**). At the above thresholds, the sensitivity was 77.4% and the specificity 6 was8% in training group, while in validation group, the sensitivity was 88.1% and the specificity was 57.4% (**Figures 3B, D**). The C-index of the predictive model was 0.774 (95% CI: 0.757-0.791) in the training group and 0.770 (95% CI: 0.731-0.809) in the validation group (**Table 4**). These results suggested that the model exhibited fair performance.

**Table 4:** The C-index and AUC of the nomogram in the training and validation groups.

Outcome	Model	Training cohort		Validation cohort	
		C-index	AUC	C-index	AUC
PTD	Female BMI + Smoking + Uterine factors + Cervical factors + Ovulatory factors + No. of embryo transfer + Blastocyst transfer + FET + Gestational sacs + PP +PA + HDP + PROM+ Male fetus	0.774 (0.757-0.791)	0.774	0.770 (0.731-0.809)	0.770

PTD, preterm delivery; BMI, body mass index; FET, frozen-thawed embryo transfer; PP, placenta previa; PA, placenta abruption; HDP, hypertensive of pregnancies; PROM, premature rupture of membrane; C-index, concordance index; AUC, area under ROC.

## Discussion

PTD is one of serious obstetric complications, which accounts for 11% of pregnancies all over the world (23). A growing number of reports have indicated that PTD was more common in IVF/ICSI pregnancies than natural pregnancies (7). There is still no effective predictive model based on risk factors to predict the incidence of PTD in IVF singleton pregnancies.

In this study, we used multiple logistic regression analysis to identify significant risk factors related with PTD in the training group, including maternal obesity, smoking, uterine factors, cervical factors, ovulatory factors, DET, blastocyst transfer, FET, double gestational sacs, obstetric complications (PP, PA, HDP, and PROM), and a male fetus. All of the above factors were utilized to develop the prognostic model, which was visualized by a nomogram. Good discrimination and calibration were shown in this model, as assessed by the AUC, C-index, and calibration plots in both the training and validation groups. Hence, this nomogram model for predicting PTD in IVF/ICSI singleton live births had fair performance and might hold promise for clinical use.

We demonstrated that PTD was affected by inherent female characteristics and infertility factors, such as BMI, smoking, uterine factors, cervical factors, and ovulatory factors. The findings of our study are in accordance with previous studies. For example, Cnattingius et al. found that overweight women in Sweden had a higher risk of PTD (24). Moreover, another systematic review also supported the above result, suggesting that there was an increasing risk of PTD for women with obesity (25). The intrauterine environment is critical to the normal function of the placenta, and the fetus develops. Maternal obesity might provide a chronic inflammatory environment, in which the development of the fetus is impaired, consequently leading to PTD (26, 27). Moreover, many studies have showed that smoking was the most important risk factor for PTD (28, 29). Therefore, women after IVF/ICSI treatment need to control their weight and quit the smoking habit to prevent the occurrence of PTD.

In terms of infertility factors, uterine factors, such as septate uterus, can cause an increase in the risk of PTD (30). Besides, it is well-known that cervical factors, such as cervical shortening (a cervical length of less than 25 mm) and cervical incompetence, are associated with PTD (6, 31). In this occasion, progesterone support is available for women with short cervix, while cervical cerclage is required for women with cervical insufficiency (32, 33). In addition, a large study enrolling 635604 IVF/ICSI cycles in the UK reported that infertile women with ovulatory disorders also had a greater chance of having a preterm baby after adjusting for confounding factors (34). Ovulatory disorder is a cause of infertility with polycystic ovary syndrome (PCOS) (35). A higher risk of PTB after fresh IVF/ICSI cycles was observed in women with PCOS than in controls (36). One of the possible explanations may be the existence of hyperandrogenism in the environment (36). It was reported that the remodeling and ripening of the cervix could be affected by androgens; thus, the rate of PTD rose (36). For infertility women with polycystic ovary and hyperandrogenism, androgen reduction therapy should be performed first, followed by ovulation induction, and pregnancy monitoring also needs to be strengthened to prevent PTD.

In addition, embryo factors may also affect PTD, such as No. of embryo transferred, blastocyst, and frozen-thawed embryo. The results showed that infertile women with SET had a lower risk of PTD than those with DET, which was in line with previous studies (37). Hence, reproductive doctors should encourage infertility women with IVF/ICSI treatment to undergo SET. Moreover, blastocyst transfer was a risk factor for PTD in comparison with cleavage transfer, which was in line with the result of a previous study (38). As for the underlying mechanism, the *in vitro* culture might affect the potential genetic or epigenetic on the trophectoderm cells; thus, the implantation and placentation were different which might cause higher incidence of PTD (38). Besides, there was a higher risk of PTD in cycles with frozen-thawed embryos than that with fresh embryos, which was accordance with a previous study (11). The possible reasons are still unknown.

During pregnancy, the incidence of PTD was higher in women with two gestational sacs and obstetric complications. Women would be more likely to be born a preterm infant if there were double gestational sacs during pregnancy but had a singleton live birth. This phenomenon is also called vanishing twin syndrome (VTS) (39). Other researchers discovered that a higher risk of PTB only occurred when VTS occurred after gestational week 14, suggesting that it was dependent on the timing of vanishing twins (40). Therefore, women who develop VTS after 14 weeks’ gestation need to strengthen pregnancy care and fetal monitoring. In addition, regardless of the methods of conception, obstetric complications, such as PP, PA, HDP, and PROM, are all related to a high incidence of PTD (6, 41). For the above different obstetric complications, different treatment measures should be performed to reduce the occurrence of PTD. If PTD is inevitable, timely treatment should be carried out to ensure the safety of mother and child.

Interesting, there was a higher risk for PTD in male fetuses compared with female fetuses. Although the underlying mechanisms are still not clear, the different trophoblast cells may explain it. Trophoblast in pregnancies with male fetuses may produce more pro-inflammatory cytokines; thus, intrauterine inflammation may cause a high incidence of PTD in male fetuses (42).

However, we did not find an association between IVF/ICSI treatment and PTD, which is different from the findings of some studies (10, 12). For instance, data available by Pinborg et al. showed that there was a lower risk of PTD in ICSI singletons than in IVF singletons, indicating that the fertilization procedure might have an effect on this perinatal outcome (12). Nevertheless, other work described that the reason behind this difference was that the high risk of PTD in IVF singletons might be secondary to female inherent risk factors instead of IVF itself (9). Although the type of IVF/ICSI treatment might not affect PTD, we found women with GnRH-antagonist protocol had higher risk to deliver a preterm baby, while women with GnRH-agonist protocol had lower risk. Zhu et al. also observed a higher PTD rate in GnRH-antagonist group (9.0%) than that in GnRH-agonist (4.0%) after propensity matching, but there was no significant difference (43). Hence, it’s possible that our results may be due to women’s inherent clinical characteristics, such as advanced age.

As for the advantages of our research, the first is the large sample size. 11513 cycles resulting in singleton births were included for analyzing. Besides, important data regarding clinical characteristics (smoking, the causes of infertility, and obstetric complications) and IVF/ICSI-ET procedures (different stimulation protocols, type of fertilization, and type of embryo transfer) were available in our study. Last but not least, to the best of our knowledge, this was the first study to construct a nomogram model for predicting the incidence of PTD in women after IVF/ICSI. It might also help doctors identify infertile women who are at higher risk of PTD. Therefore, those patients who had obesity, a smoking habit, uterine factors, cervical factors, ovulatory factors, DET, blastocyst transfer, FET, VTS, obstetric complications, or a male fetus after IVF/ICSI should be informed of the possible high rate of PTD. They are also encouraged to actively cooperate with prenatal examinations and care to reduce the occurrence of this poor neonatal outcome.

Nevertheless, several limitations in our study also need to be addressed. First, this study was carried out in a single reproductive center and lacks external validation in other centers; thus, there might be inevitable bias. Additionally, potential biases cannot be excluded as it is a retrospective study. Third, all possible related factors of PTD might not be covered in this analysis. For example, some risk factors, including women smoking, previous obstetric history, genital tract infection, etc., may be significant with PTD as well, yet the data were not available. Last, two subtypes (spontaneously preterm birth and iatrogenic preterm birth) of PTD cannot be separated by us. Actually, there are different risk factors between these two subtypes.

In summary, our study explored the risk factors for PTD in women with IVF/ICSI singleton live births and found that the incidence of PTD rose when it comes to maternal obesity, infertility factors, blastocyst transfer, FET, DET, VTS, obstetric complications, or a male fetus. Furthermore, the nomogram for visualizing the predictive model was built by us and demonstrated good discrimination and calibration to some extent. Hence, it might be of great value for clinical use. Notwithstanding that, more prospective studies need to be conducted to investigate risk factors for PTD in IVF/ICSI singleton live birth and validate our predictive tool.

## Data availability statement

The original contributions presented in the study are included in the article/**Supplementary Material**. Further inquiries can be directed to the corresponding authors.

## Ethics statement

The study was approved by the Medical Ethics Committee of Tongji Hospital, Tongji Medical College, affiliated Huazhong University of Science and Technology (TJ-IRB20220624).

## Author contributions

ZL contributed to the design of study. ZL, LC, JL, XH, YL, and LS collected data. ZL performed data analysis. ZL drafted the manuscript, which was revised by CL, KQ, CS, and HZ. All authors contributed to the article and approved the submitted version.
