# Supplementary material for: Nomogram for predicting the risk of preterm delivery after IVF/ICSI treatment: an analysis of 11513 singleton births
Source: Front Endocrinol (Lausanne). 2023 May 18;14:1065291. doi: 10.3389/fendo.2023.1065291 (PMC10233110; doi:10.3389/fendo.2023.1065291)
Supplement: Supplementary file 1 [file Table_1.docx]

**Table S1** Baseline characteristics of patients with singleton live birth between PTD and non-PTD groups in the fresh cycles.

| Variables | Non-PTD  n = 6034 | PTD  n = 450 | P value |
| --- | --- | --- | --- |
| Female age, y |  |  |  |
| <35  ≥35 | 5187 (86.0%)  847 (14.0%) | 366 (81.3%)  84 (18.7%) | .007* |
| Female BMI, kg/m^2^ |  |  |  |
| ≤24  >24 | 4033 (66.8%)  2001 (33.2%) | 279 (62.0%)  171 (38.0%) | .036* |
| Smoking | 60 (1.0%) | 39 (8.7%) | <.001* |
| Duration of infertility^#^, y | 3.00 (2.00) | 3.00 (3.00) | .324 |
| AFC |  |  | .246 |
| 5-24  <5  ≥24 | 5088 (84.3%)  244 (4.0%)  702 (11.6%) | 366 (81.3%)  22 (4.9%)  62 (13.8%) |  |
| Baseline FSH^#^, mIU/ml | 7.29 (2.3) | 7.28 (2.24) | .805 |
| Type of infertility |  |  | .211 |
| Primary  Secondary | 3904 (64.7%)  2130 (35.3%) | 278 (61.8%)  172 (38.2%) |  |
| Factors of infertility |  |  |  |
| Tubal  Pelvic  Endometriosis  Uterine  Cervical  Ovulatory  DOR  Male  Unexplained | 3344 (55.4%)  1211 (20.1%)  721 (11.9%)  1061 (17.6%)  32 (0.5%)  1267 (21.0%)  778 (12.9%)  2270 (37.6%)  309 (5.1%) | 236 (52.4%)  96 (21.3%)  56 (12.4%)  94 (20.9%)  13 (2.9%)  117 (26.0%)  79 (17.6%)  160 (35.6%)  19 (4.2%) | .221  .519  .755  .077  <.001*  .012*  .005*  .383  .401 |
| Stimulation protocols |  |  | <.001* |
| GnRH-a ultralong  depot GnRH-a  GnRH-a long  GnRH-ant | 61 (1.0%)  2725 (45.2%)  1715 (28.4%)  1533 (25.4%) | 5 (1.1%)  210 (46.7%)  85 (18.9%)  150 (33.3%) |  |
| Average dose of Gn^#^, IU/day | 225.00 (98.75) | 225.00 (114.96) | .064 |
| E_2_ on hCG^#^, pg/ml | 2328.50 (1717) | 2132.50 (1635.75) | .029* |
| P on hCG, pg/ml^&^ | 0.78 ± 0.37 | 0.76 ± 0.34 | .168 |
| EMT on hCG, mm |  |  | .028* |
| ≤7  >7 | 74 (1.2%)  5960 (98.8%) | 11 (2.4%)  439 (97.6%) |  |
| No. of oocyte retrieved |  |  |  |
| 9-15  <9  >15 | 2898 (48.0%)  1782 (29.5%)  1354 (22.4%) | 206 (45.8%)  143 (31.8%)  101 (22.4%) | .562 |
| Treatment type  IVF  ICSI  IVF+RICSI | 3950 (65.5%)  1766 (29.3%)  318 (5.3%) | 301 (66.9%)  127 (28.2%)  22 (4.9%) | .818 |
| No. of embryo transferred |  |  | .072 |
| 1 | 3494 (57.9%) | 241 (53.6%) |  |
| 2 | 2540 (42.1%) | 209 (46.4%) |  |
| Blastocyst transfer | 515 (8.5%) | 48 (10.7%) | .121 |
| High-quality embryo transfer | 5385 (89.2%) | 397 (88.2%) | .501 |
| Gestational sacs  1  2 | 5638 (93.4%)  396 (6.6%) | 392 (87.1%)  58 (12.9%) | <.001* |
| PP | 164 (2.7%) | 47 (10.4%) | <.001* |
| PA | 7 (0.1%) | 24 (5.3%) | <.001* |
| GDM | 308 (5.1%) | 39 (8.7%) | .001* |
| HDP | 110 (1.8%) | 37 (8.2%) | <.001* |
| PROM | 41 (0.7%) | 56 (12.4%) | <.001* |
| Neonatal gender |  |  | .044* |
| Male | 3244 (53.8%) | 264 (58.7%) |  |
| Female | 2790 (46.2%) | 186 (41.3%) |  |
| Birth weight^#^, g | 1850.00 (1550.00) | 1325.00 (375.00) | <.001* |

**Table notes:** PTD: preterm delivery. BMI: body mass index. AFC: antral follicles count. FSH: follicle-stimulating hormone. DOR: diminished ovarian reserve. GnRH-a: gonadotropin releasing hormone agonist. GnRH-ant: GnRH antagonist. Gn: gonadotropin. E_2_: estradiol. P: progesterone. EMT: endometrial thickness. hCG: human chorionic gonadotrophin. IVF: *in vitro* fertilization. ICSI: intracytoplasmic sperm injection. RICSI: rescue ICSI. PP: placenta previa. PA: placenta abruption. GDM: gestational diabetes mellitus. HDP: hypertensive of pregnancies. PROM: premature rupture of membrane. #: data are presented as median (IQR). &: data are presented as mean ± SD. * P<0.05.

**Table S2** Baseline characteristics of patients with singleton live birth between PTD and non-PTD groups in the FET cycles.

| Variables | Non-PTD  n = 4303 | PTD  n = 726 | P value |
| --- | --- | --- | --- |
| Female age, y |  |  | .552 |
| <35  ≥35 | 3577 (83.1%)  726 (16.9%) | 597 (82.2%)  129 (17.8%) |  |
| Female BMI, kg/m^2^ |  |  | <.001* |
| ≤24  >24 | 3490 (81.1%)  813 (18.9%) | 548 (75.5%)  178 (24.5%) |  |
| Smoking | 40 (0.9%) | 10 (1.4%) | .261 |
| Duration of infertility^#^, y | 3.00 (2.00) | 3.00 (2.00) | .704 |
| AFC |  |  | .245 |
| 5-24  <5  ≥24 | 3900 (90.6%)  196 (4.6%)  207 (4.8%) | 644 (88.7%)  38 (5.2%)  44 (6.1%) |  |
| Baseline FSH^#^, mIU/ml | 7.10 (2.22) | 7.01 (2.23) | .175 |
| Type of infertility |  |  | .040* |
| Primary  Secondary | 2980 (69.3%)  1323 (30.7%) | 475 (65.4%)  251 (34.6%) |  |
| Factors of infertility |  |  |  |
| Tubal  Pelvic  Endometriosis  Uterine  Cervical  Ovulatory  DOR  Male  Unexplained | 2149 (49.9%)  722 (16.8%)  243 (5.6%)  865 (20.1%)  28 (0.7%)  1134 (26.4%)  485 (11.3%)  1644 (38.2%)  296 (6.9%) | 386 (53.2%)  136 (18.7%)  62 (8.5%)  194 (26.7%)  6 (0.8%)  202 (27.8%)  82 (11.3%)  250 (34.4%)  43 (5.9%) | .108  .195  .003*  <.001*  .755  .407  .985  .052  .342 |
| Endometrial preparation |  |  | .200 |
| Natural  HRT | 266 (6.2%)  4037 (93.8%) | 54 (7.4%)  672 (92.6%) |  |
| EMT, mm |  |  | .391 |
| ≤7  >7 | 107 (2.5%)  4196 (97.5%) | 22 (3.0%)  704 (97.0%) |  |
| Treatment type  IVF  ICSI  IVF+RICSI | 2835 (65.9%)  1231 (28.6%)  237 (5.5%) | 505 (69.6%)  186 (25.6%)  35 (4.8%) | .152 |
| No. of embryo transferred |  |  | <.001* |
| 1 | 3127 (72.7%) | 378 (52.1%) |  |
| 2 | 1176 (27.3%) | 348 (47.9%) |  |
| Blastocyst transfer | 3888 (90.4%) | 670 (92.4%) | .099 |
| High-quality embryo transfer | 3167 (73.6%) | 489 (67.4%) | <.001* |
| Gestational sacs  1  2 | 3837 (89.2%)  466 (10.2%) | 567 (78.1%)  159 (21.9%) | <.001* |
| PP | 93 (2.2%) | 43 (5.9%) | <.001* |
| PA | 32 (0.7%) | 48 (6.6%) | <.001* |
| GDM | 213 (5.0%) | 30 (4.1%) | .342 |
| HDP | 106 (2.5%) | 81 (11.2%) | <.001* |
| PROM | 13 (0.3%) | 48 (6.6%) | <.001* |
| Neonatal gender |  |  | .023* |
| Male | 2342 (54.4%) | 428 (59.0%) |  |
| Female | 1961 (45.6%) | 298 (41.0%) |  |
| Birth weight^#^, g | 3400.00 (580.00) | 2450.00 (758.00) | <.001* |

**Table notes:** PTD: preterm delivery. FET: frozen-thawed embryo transfer. BMI: body mass index. AFC: antral follicles count. FSH: follicle-stimulating hormone. DOR: diminished ovarian reserve. HRT: hormone replacement cycles. EMT: endometrial thickness. IVF: *in vitro* fertilization. ICSI: intracytoplasmic sperm injection. RICSI: rescue ICSI. PP: placenta previa. PA: placenta abruption. GDM: gestational diabetes mellitus. HDP: hypertensive of pregnancies. PROM: premature rupture of membrane. #: data are presented as median (IQR). * P<0.05.
